# Regional variations in T1 in the healthy left ventricle

**DOI:** 10.1186/1532-429X-15-S1-E43

**Published:** 2013-01-30

**Authors:** Hazel D Rovno, Scott Akers, Harold Litt

**Affiliations:** 1 Radiology, University of Pennsylvania, Philadelphia, PA, USA; 2 Radiology, VAMC, Philadelphia, Philadelphia, PA, USA

## Background

T1 mapping is a newer technique for characterizing the myocardium, however regional variations in the healthy left ventricle are not yet fully explored, complicating interpretation of observed pathologic differences.

## Methods

We assessed regional variation in T1 in the septum of patients with no left ventricular pathology. An adjunct phantom study was performed to assess whether location closer to a surface coil affected T1. Main study: Retrospective. 40 patients whose cardiac MRI showed no left ventricular disease were identified. Patients were imaged on a Siemens Avanto 1.5T scanner with a 32-channel cardiac coil. All patients had a 4-chamber TI scout approximately 12 minutes post injection of 0.14 mmol/kg gad-BOPTA. QMASS (Medis, NL) was used to generate T1 maps and measurements obtained in small regions of interest throughout the septum. Basal, middle third, and apical regions were evaluated in all 40; a subset of 15 patients had an additional roi placed halfway between the middle and apical ROIs. Adjunct study: TI scout and MOLLI images of a liquid phantom (homogeneous T1 of approximately 315 ms, NiSO_4_ in H_2_O, NaCl) phantom were obtained at several simulated heart rates.

## Results

Measured T1 values in the septum were: Basal septum 384 ±46 ms (287-484). Midventricular septum 388 ±44 ms (271-477). ¾ distance base to apex along the septum, 356 ±56 ms (233-456). Apical septum 346 ±52 ms (197-459).

By 2-tailed paired t-test, T1 of the basal vs. mid-ventricular septum was not significantly different (p=0.33). However, mid-ventricle was different from ¾ (p=0.008) and apical septum (p<0.0001) with paired t-tests. Unpaired t-test comparison of midventricular to apical septum showed p=0.0002 likelihood that the difference was due to chance.

In the homogeneous T1 liquid phantom, the intra-study T1 difference (i.e., heterogeneity by region) derived from TI scout imaging was 7-15 ms, from MOLLI 10-35 ms. There was thus less intra-study regional variation using the TI scout. However, whole-phantom T1 measured by TI scout was more affected by heart rate (288-314 ms) than MOLLI (314-316 ms).

## Conclusions

1) We found a gradient of T1 over the distal half of the septum. The findings may reflect a systematic artifact, or a true regional difference in septal myocardial T1. In either case, the implication is that exact slice location may be important when performing T1 mapping.

Work is currently underway to explore whether this finding reflects a true difference in regional myocardium, or an artifact. Possible explanations include: regional differences in extracellular volume, collagen, or fat content of the normal septum, effects of fiber orientation, regional perfusion / gadolinium kinetics, volume averaging of blood pool related to curvature of the distal septum, motion effects, and susceptibility artifacts related to proximity to lung vs mediastinal tissues.

2) Phantom studies excluded the possibility that this variation is due simply to the fact that the apex is closer to the receiver coil.

## Funding

none

**Figure 1:**
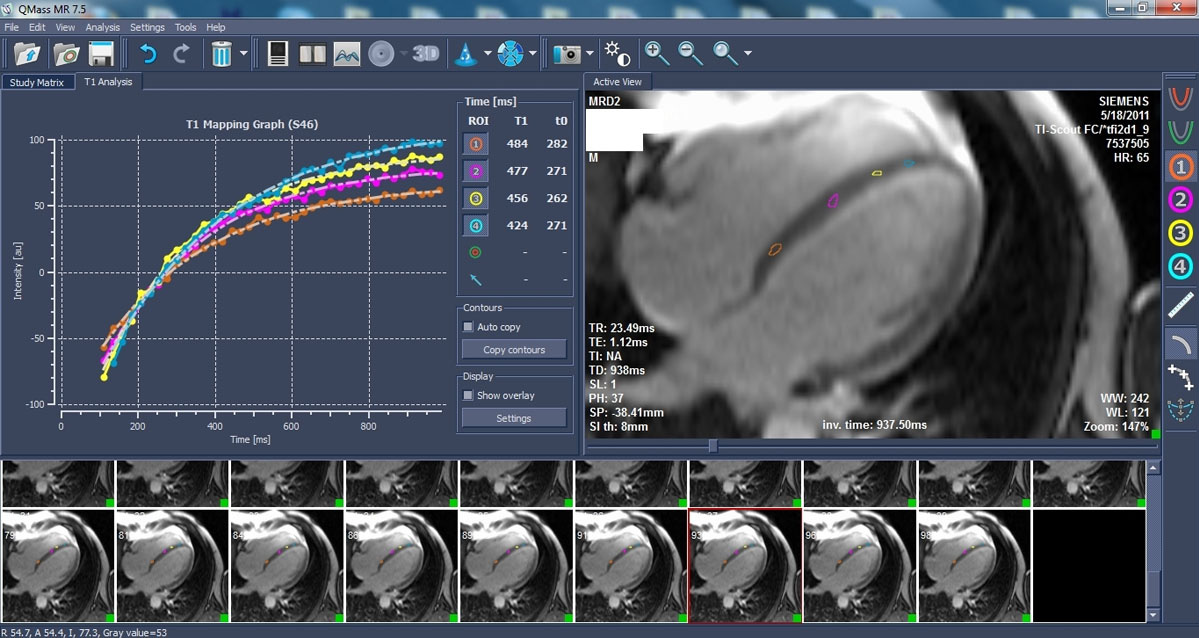
Sample QMASS output for the septal regions. Each color-coded roi is reflected as a T1 fitting curve. Because of motion over the cardiac cycle, roi were manually adjusted to cover comparable areas.

**Figure 2:**
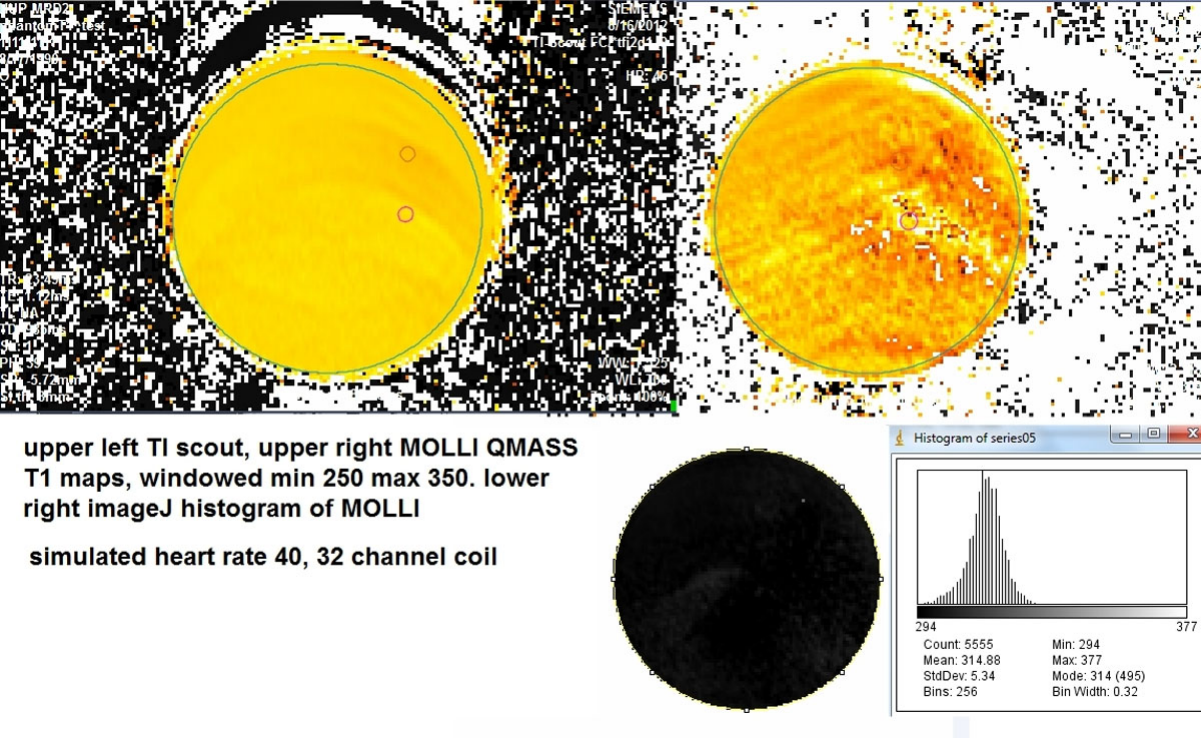
Left - TI scout of axial slice through cylindrical liquid phantom. Right - MOLLI of same slice, same simulated heart rate (40 bpm), same windowing. Upper left and right are T1 maps from QMASS. Lower right is Siemens reconstruction of the MOLLI sequence with a corresponding histogram (from imageJ). Note the superior intrastudy homogeneity of the TI scout T1 map.

